# Lipid Extracted Microalgal Biomass Residue as a Fertilizer Substitute for *Zea mays* L.

**DOI:** 10.3389/fpls.2015.01266

**Published:** 2016-01-20

**Authors:** Rahulkumar Maurya, Kaumeel Chokshi, Tonmoy Ghosh, Khanjan Trivedi, Imran Pancha, Denish Kubavat, Sandhya Mishra, Arup Ghosh

**Affiliations:** ^1^Salt and Marine Chemicals Division, CSIR-Central Salt and Marine Chemicals Research InstituteBhavnagar, India; ^2^Academy of Scientific and Innovative Research, CSIR-Central Salt and Marine Chemicals Research InstituteBhavnagar, India; ^3^Wasteland Research Division, CSIR-Central Salt and Marine Chemicals Research InstituteBhavnagar, India

**Keywords:** *Chlorella*, *Lyngbya*, fertilizer, de-oiled microalgae, maize, soil properties, photosynthesis

## Abstract

High volumes of lipid extracted microalgal biomass residues (LMBRs) are expected to be produced upon commencement of biodiesel production on a large scale, thus necessitating its value addition for sustainable development. LMBRs of *Chlorella variabilis* and *Lyngbya majuscula* were employed to substitute the nitrogen content of recommended rate of fertilizer (RRF) for *Zea mays* L. The pot experiment comprised of 10 treatments, i.e., T1 (No fertilizer); T2 (RRF-120 N: 60 P_2_O_5_: 40 K_2_O kg ha^−1^); T3 to T6—100, 75, 50, and 25% N through LMBR of the *Chlorella* sp., respectively; T7 to T10—100, 75, 50, and 25% N through LMBR of *Lyngbya* sp., respectively. It was found that all LMBR substitution treatments were at par to RRF with respect to grain yield production. T10 gave the highest grain yield (65.16 g plant^−1^), which was closely followed by that (63.48 g plant^−1^) under T5. T10 also recorded the highest phosphorus and potassium contents in grains. T4 was markedly superior over control in terms of dry matter accumulation (DMA) as well as carbohydrate content, which was ascribed to higher pigment content and photosynthetic activity in leaves. Even though considerably lower DMA was obtained in *Lyngbya* treatments, which might have been due to the presence of some toxic factors, no reduction in grain yield was apparent. The length of the tassel was significantly higher in either of the LMBRs at any substitution rates over RRF, except T6 and T7. The ascorbate peroxidase activity decreased with decreasing dose of *Chlorella* LMBR, while all the *Lyngbya* LMBR treatments recorded lower activity, which were at par with each other. Among the *Chlorella* treatments, only T5 recorded significantly higher values of glutathione reductase activity over RRF, while the rest were at par. There were significant increases in carbohydrate and crude fat, respectively, only in T4 and T3 over RRF, while no change was observed in crude protein due to LMBR treatments. Apparently, there was no detrimental effect on soil properties, suggesting that both the LMBRs can be employed to reduce the usage of chemical fertilizers, thus promoting maize crop production in a sustainable manner.

## Introduction

The effect of climate change due to the incessant burning of fossil fuels is now being felt across the globe (Ghosh, 2012) which has necessitated the search for alternate sources of cleaner energy (Ghosh et al., 2007; Ghosh, 2014). In the last few years, there has been intensive research on microalgae as it is an attractive feedstock for biofuel production (Chisti, 2007). Microalgae have been reported to have much higher primary productivity as compared to other terrestrial plants having biofuel potential (Mata et al., 2010). Processes for conversion of microalgal oil to biodiesel have already been developed (Mishra et al., 2012) and it is expected that once the microalgal cultivation technology is sufficiently optimized, adequate feedstock would be available from large scale cultivation for biofuel production. During algal biodiesel production, lipid is extracted from microalgal biomass through suitable solvent and converted to biodiesel through trans-esterification process. After lipid extraction, a huge amount of de-oiled microalgal biomass remains left.

Algae biomass is a complex material comprising of carbohydrates, lipids, proteins, and several potentially interesting molecules. The biofuel sector is specifically interested in carbohydrates (mainly C6) and lipids (mainly C16–C22), the amount of which could vary as a function of the algae species, strains, and the cultivation methods adopted (Chiaramonti et al., 2015). The current worldwide algae biomass production has been projected to be at more than 20,000 dry tons per year (Verdelho, 2013; Zittelli et al., 2013a,b; Chiaramonti et al., 2015). However, the algae market is expected to grow as the constraints of algal production are overcome and the cost of production is reduced. As per Studt (2010) and Rodolfi et al. (2009), the potential oil yield from microalgae on equal acreage basis is 5–20 times higher than that of oil from palm species. Assuming a conservative figure of 25% extractable oil from microalgal biomass (Chisti, 2007), for every metric ton of biodiesel manufactured, three times the amount of lipid extracted microalgal biomass residue (LMBR) will be produced. To make the biodiesel sustainable, it is also necessary to valorize such biomass for various applications, thus also offsetting the cost of biodiesel. Such value addition of LMBRs may be by its use as feed and fertilizer; fermentation to bio-methane and bio-ethanol; as a nutrient source for organisms; thermo-chemical conversion into various fuels and chemicals and as biosorbents for removal of dye and heavy metals from wastewater (Mata et al., 2010; Scott et al., 2010; Rashid et al., 2013; Maurya et al., 2014). Depending on the species, LMBRs are rich in protein and therefore rich in nitrogen content besides the presence of other essential plant macro- and micro-nutrients. Because of low carbon-nitrogen ratio, it cannot be utilized directly for bio-methane production, although such biomass can be utilized as animal feed, fertilizer, or nutrient source for organisms (Mata et al., 2010; Bryant et al., 2012; Gao et al., 2012). There are reports that shed light on the use of microalgae as a potential source of nutrients and bioactive compounds that can be utilized for sustainable plant production either directly as an inoculum (Metting and Rayburn, 1983; Grzesik and Romanowska-Duda, 2014), as unprocessed dried algae (Mulbry et al., 2005), and sonicated biomass (Grzesik and Romanowska-Duda, 2015). However, no such studies have been reported yet that validate the use of LMBRs as a source of plant nutrients.

Urea, which is the most commonly used chemical nitrogenous fertilizers, is produced through very well-known Haber-Bosch process which rely on fossil fuels (Vance, 2001; Pfromm et al., 2011). Excessive use of chemical fertilizers is responsible for global warming, for example, assuming a recommended rate of 150:60:40 kg N:P_2_O_5_:K_2_O hectare^−1^ applied through urea, di-ammonium phosphate (DAP) and muriate of potash (MOP) for the cultivation of maize, ~599 kg CO_2_ equivalents are produced on account of fertilizer production and their transport (Singh et al., 2015). Using carbon rich residues as a chemical fertilizer substitute may also have other benefits such as improved soil health, stability of soil aggregates, soil water retention, carbon sequestration, and prevention of nutrient losses (Metting and Rayburn, 1983; Anand et al., 2015). Enhancing the productivity of crops in a sustainable manner without adversely affecting soil health should be the focus of the approaches to meet the increasing food demand (Singh et al., 2015).

In the present study, lipid-extracted biomass residues of two microalgal species, namely, *Chlorella variabilis* (ATCC PTA 12198) and *Lyngbya majuscula* have been evaluated for its nitrogen substitution potential for maize (*Zea mays* L.—a heavy feeder of nutrients) crop in a pot experiment. Different N substitution levels were used to evaluate their effects on crop growth, yield, quality, and soil properties.

## Materials and methodology

### LMBR preparation and characterization

Initially, 1 kg biomass of each algae was taken for the lipid extraction. The lipid was extracted in soxhlet extractor using 3 L n-hexane per kg dry biomass. The extraction time of five cycles was 4 h in total. At the end of extraction, lipid, hexane, and de-oiled biomass were recovered. The de-oiled biomass was sundried for a day and powdered by grinding in mixture grinder (Boss Cyclone B219, Boss Home Appliances, Mumbai, India). The chemical composition of *Chlorella* and *Lyngbya* LMBR has been presented in Table 1. TVS and ash were obtained by subjecting the ovendried biomass in a muffle furnace at 575 ± 25°C (van Wychen and Laurens, 2013). Carbon, hydrogen, nitrogen and sulfur were analyzed using a CHNS analyzer (Perkin-Elmer Model 2 400, USA). Na and K was analyzed after wet ashing the LMBR samples with ternary acid mixture (Jackson, 1973), followed by their estimation using flame photometer (Toth et al., 1948); other micro and macro elements were analyzed by the X-Ray Fluorescence analyzer (Bruker AXS, S4 Pioneer, Billerica, Massachusetts, USA). Crude protein was calculated from nitrogen content using a multiplication factor of 6.25 (Jones, 1941).

**Table 1:** **Composition of LMBRs from *Chlorella* and *Lyngbya***.

**Parameters**	**LMBR**	
	***Chlorella***	***Lyngbya***
TVS (%)	67.66±0.071	55.82±2.58
Ash (%)	32.34±0.071	40.73±1.57
Crude protein (%)	36.31±0.98	24.75±0.28
C (%)	28.36±2.51	23.40±1.56
H (%)	4.75±0.32	4.42±0.28
N (%)	5.810±0.157	3.960±0.045
S (%)	0.27±0.04	0.43±0.03
P (%)	0.273±0.072	0.097±0.004
K (%)	0.634±0.031	0.579±0.044
Na (%)	1.466±0.016	0.183±0.005
Ca (%)	0.017±0.002	0.007±0.001
Mg (%)	0.07±0.01	0.037±0.004
Fe (%)	1.84±0.19	6.61±1.21
Mn (%)	0.05±0.008	0.27±0.08
Cu (PPM)	6±1.35	6±1.00
Zn (PPM)	31±3.88	24±4.36
Cl (PPM)	69±8.56	64±9.27

### Pot experiment

The experiment was carried out during *Rabi* season in 2013–14 (i.e., cold months during November to February) at pot culture facility of CSIR-CSMCRI, Bhavnagar, Gujarat, India (21°44′57.6″N latitude, 72°08′39.3″E longitude). The experimental soil was sandy clay loam in texture, slightly alkaline (pH: 8.08 ± 0.03) and non-saline (0.41 dS m^−1^). The soil was low in organic carbon (0.21 ± 0.05%), medium in available N (0.2 ± 0.06 g kg^−1^), and high with respect to available P (0.027 ± 0.004 g kg^−1^) and available K (0.30 ± 0.01 g kg^−1^). The experiment was carried out in pots with 35 kg soil in each of them. The experiment was laid out in completely randomized design (CRD) comprising of 10 treatments including control (recommended rate of fertilizers through chemical means) and absolute control receiving no nutrients at all. Random number table was used for randomization. Each of the 10 treatments had five replicates, each represented by an individual pot having one plant. The recommended rate of fertilizer (RRF) application was 120:60:40 kg N:P_2_O_5_:K_2_O hectare^−1^ supplied through urea, single super phosphate (SSP), and MOP, respectively. The test variety employed was F1 hybrid sweet corn, variety: Sugar-75 (Syngenta India Ltd.). This variety has very good plant vigor, plant height and is recommended for winter sowing. The maturity of the plant is about 90 days. The variety has long uniform cylindrical cobs, golden yellow kernels, excellent tip filling, high yield, and very sweet taste (16% total soluble sugar).

The treatments T_1_ and T_2_ represented absolute control and RRF, respectively. The treatments T_3_–T_6_ represented substitution of 100, 75, 50, and 25% N, respectively, through *Chlorella* derived LMBR. Similarly, treatments T_7_–T_10_ represented substitution of 100, 75, 50, and 25% N, respectively, through *Lyngbya* derived LMBR. The remaining proportion of nitrogen (i.e., 0, 25, 50, and 75% of RRF) in the respective treatments was supplemented through urea fertilizer (containing 46% N) to accomplish RRF in all the treatments except T1. The phosphorus and potassium inadvertently supplied through biomass were calculated and remaining dose was supplied through SSP and MOP to accomplish the RRF in all the treatments except T1. The biomass and chemical fertilizers were applied to the soil on the day of sowing according to the respective treatments. The crop was harvested at maturity (110th day after sowing).

### Recording of growth parameters

Observations on growth parameters [plant height at 20, 40, 60, and 80 days after sowing (DAS), dry matter accumulation (DMA) in leaves, stem, and roots at harvest], grain yield and yield attributes (100-grain weight, number of kernel (grain) rows per cob and number of kernels per row) were recorded. Chlorophyll index of leaves at 20, 40, 60, 80, and 100 DAS was measured using Chlorophyll meter (Opti-Sciences, CCM-200). Photosynthetic rate was measured at 30 and 60 DAS by infrared gas analyzer (LI-6400XT, portable photosynthesis system, LI-COR, USA).

### Enzyme assays

Enzyme assays were carried at 60 DAS, which corresponded to 10 days pre-tasseling. Any kind of environmental stresses like nutrient or moisture deficiency at this late vegetative growth stage greatly affects the crop performance and therefore this time was chosen to assess any stress response due to the different nutrient management treatments (McWilliams et al., 1999). For carrying out enzyme assays, leaf tissues (0.1 g fresh weight) were collected at 60 DAS and homogenized using a mortar and pestle with liquid nitrogen. The tissue was then extracted with 50 mM Tris extraction buffer (pH 7.5) containing 0.2% (w/v) Triton X-100, 0.1 mM EDTA, 1 mM polymethylsulphonylfluoride, and 2 mM dithiothreitol for glutathione reductase (GR) activity. For ascorbate peroxidase (APX) assay, instead of Tris, 50 mM potassium phosphate buffer (pH 7.0) amended with 2 mM ascorbate was used for extraction. Both the homogenates were vortexed and centrifuged at 20,000 × g for 30 min at 4°C. The supernatants were collected and stored at −80°C until further analysis. Protein content was estimated according to Bradford method (1976) using bovine albumin as a standard and measured using UV–Vis spectrophotometer. The APX (EC 1.11.1.1) and GR (EC 1.6.4.2) activity were measured according to Nakano and Asada (1981) and Edwards et al. (1990), respectively. The concentration of oxidized ascorbate was calculated using an extinction coefficient of 2.8 mM^−1^cm^−1^, while GR activity was calculated using an extinction coefficient of 6.22 M^−1^cm^−1^ for NADPH. One unit of APX is defined as 1 mmol ascorbate oxidized per min per ml and expressed in units per mg protein. One unit of GR is defined as 1 μmol NADPH oxidized per min per ml at 25°C and expressed in units per mg protein.

### Analytical methods

The total nitrogen content in the grain was estimated by standard semi-micro-Kjeldahl method (Thimmaiah, 1999) and expressed in terms of crude protein using a conversion factor of 6.25 (Jones, 1941), while Na and K content was determined using flame photometry (Flame photometer 128, Systronics, India) according to the method prescribed by Jackson (1973). Phosphorus (P) content in grains was determined by the Vanado-Molybdate yellow method (Jackson, 1973) following wet digestion with HNO_3_–HClO_4_ (10:4) di-acid mixture. Carbohydrate in grains was estimated by the method of Dubois et al. (1956). Fat content in grains was determined using petroleum ether (at 70°C) in a Soxhlet apparatus (AACC, 1987)

The available N in soil was analyzed by alkaline permanganate method (Subbiah and Asija, 1956). The available K in soil was extracted by neutral normal ammonium acetate method (Hanway and Heidel, 1952) and available P by sodium bicarbonate as per Olsen et al. (1954) and estimated by flame photometer (Flame photometer 128, Systronics, India) and spectrophotometer (Varian Cary-50 Bio, Varian Inc., USA), respectively. Organic carbon was analyzed according to the method of Walkley and Black (1934).

### Statistical analysis

The treatments were subjected to statistical analysis by analysis of variance (ANOVA) using InfoStat statistical software V 2012 (Di Rienzo et al., 2011). *Post-hoc* comparison of means was carried out using Fisher's Least Significant Difference (LSD) at the probability level of 5% and presented in the tables.

## Results

### Effects on growth parameters of maize

The plant heights recorded in different treatments at 20, 40, 60, and 80 DAS are presented in Table 2. The lowest plant height was recorded at all the growth stages under the absolute control treatment where no fertilizer was applied (T1). Compared to RRF, the treatment receiving N nutrition equally through LMBR from *Chlorella* and chemical source (T5) gave an initial fillip to the plant height as early as 20 DAS that was maintained significantly till 80 DAS (*P* < 0.001). However, all the treatments employing LMBR from *Lyngbya* were at par with RRF with respect to plant height at all the days of observations, except T8 at 60 DAS; however, there was no change in the number of nodes due to any of the treatments. Interestingly, the length of the tassels formed by applying either of the LMBRs at any of the substitution rates was markedly higher than that formed under RRF (*P* < 0.05), except that in the treatments employing the lowest level of LMBR from *Chlorella* and the highest level of that from *Lyngbya*, where were at par.

**Table 2:** **Effect of different LMBR treatments on plant height and tassel height of maize**.

**Treatments**	**Plant height (cm)**				**Tassel height(cm)**
	**20 DAS**	**40 DAS**	**60 DAS**	**80 DAS**	
T1	21.60±1.14^d^	68.40±3.85^f^	102.60±4.28^d^	164.60±2.07^e^	34.20±2.17^d^
T2	23.00±1.00^c^	76.00±2.85^bcde^	151.80±7.09^c^	199.80±4.92^bc^	37.20±1.92^cd^
T3	24.80±0.84^ab^	83.40±6.35^a^	167.60±4.98^b^	203.40±7.16^bc^	40.60±1.95^ab^
T4	23.20±1.64^c^	78.40±3.51^abc^	164.40±3.36^b^	221.80±12.01^a^	40.80±3.70^ab^
T5	26.00±1.00^a^	80.20±3.17^ab^	177.60±5.94^a^	225.40±8.82^a^	42.60±2.97^a^
T6	23.20±1.30^c^	72.60±5.86^def^	153.80±7.79^c^	185.40±7.54^d^	38.60±2.97^bc^
T7	23.40±1.14^c^	76.20±2.59^bcde^	150.20±6.76^c^	207.60±10.55^b^	39.80±1.79^abc^
T8	24.20±0.84^bc^	77.80±4.40^bcd^	163.60±4.04^b^	206.80±5.76^b^	41.60±1.14^ab^
T9	23.80±0.45^bc^	74.30±3.07^cde^	151.40±5.41^c^	194.40±2.30^cd^	41.00±2.00^ab^
T10	23.80±1.10^bc^	72.00±5.00^ef^	151.80±3.70^c^	195.80±5.63^c^	41.00±3.00^ab^

Values followed by different alphabets in columns are significantly different at p < 0.05 by LSD.

Leaf chlorophyll index and net photosynthetic rates recorded at different growth stages are tabulated in Table 3. The chlorophyll index of LMBR treated plants was significantly higher than that of RRF only at 60 DAS (*P* < 0.001) and that also in the T4 and T5 treatments in which 75 and 50% chemical N substitution was done through LMBR from *Chlorella* and in T10 which replaced 25% of N. All these three treatments were at par. The treatment T10 maintained this advantage even at 100 DAS. T5 and T10, which recorded the highest chlorophyll index at 60 DAS using *Chlorella* and *Lyngbya* LMBR, respectively, also recorded the significantly highest net photosynthetic rate (*P* < 0.001) compared to RRF for the respective LMBR at the corresponding time of observation. Both these treatments recorded higher rate of photosynthesis at the early growth stage (30 DAS) as well, however, they were at par with T4 and T6.

**Table 3:** **Effect of different LMBR treatments on leaf chlorophyll index and photosynthesis rate of maize**.

**Treatments**	**Leaf chlorophyll index**					**Photosynthesis (μmole CO_2_/m^2^leaf/day)**	
	**20 DAS**	**40 DAS**	**60 DAS**	**80 DAS**	**100 DAS**	**30 DAS**	**60 DAS**
T1	22.70±3.27^e^	31.48±4.30^c^	25.38±2.47^d^	24.86±2.93^d^	19.44±4.10^f^	25.45±1.38^e^	16.63±1.13^g^
T2	32.90±3.65^abc^	37.08±3.52^ab^	36.88±3.60^c^	47.18±3.56^a^	34.36±3.68^bc^	34.45±2.01^cd^	26.29±1.72^f^
T3	31.80±4.45^bcd^	32.72±2.22^bc^	37.12±3.17^c^	37.72±4.83^bc^	27.40±3.30^d^	33.32±0.34^d^	29.63±1.38^de^
T4	34.58±3.33^ab^	37.22±2.74^ab^	44.22±5.73^a^	36.12±2.48^bc^	36.26±2.57^ab^	41.51±4.92^a^	28.10±1.28^e^
T5	36.68±2.59^a^	35.34±2.53^bc^	47.24±5.00^a^	38.66±4.95^bc^	32.00±1.60^c^	40.95±2.16^ab^	30.41±0.62^cd^
T6	31.52±4.69^bcd^	36.42±4.22^abc^	43.48±1.50^ab^	41.68±4.25^ab^	35.80±3.68^abc^	41.21±1.83^a^	28.61±2.72^e^
T7	28.04±3.30^d^	31.44±5.23^c^	36.40±3.63^c^	37.20±4.35^bc^	24.12±2.74^de^	34.55±3.06^cd^	35.65±1.54^b^
T8	29.14±5.17^cd^	36.54±4.86^abc^	38.80±4.82^bc^	38.58±6.45^bc^	20.54±2.17^ef^	33.73±1.46^d^	31.51±0.85^c^
T9	28.20±3.18^cd^	31.62±6.63^c^	38.42±4.73^bc^	33.26±5.31^c^	27.76±2.85^d^	37.35±3.07^c^	27.87±0.19^ef^
T10	28.66±2.78^cd^	41.80±4.22^a^	45.58±4.30^a^	40.26±2.75^b^	38.52±3.58^a^	37.65±2.82^bc^	37.83±0.91^a^

Values followed by different alphabets in columns are significantly different at p < 0.05 by LSD.

The DMA in stem, leaves, and roots and their total at harvest are presented in Table 4. Supplying 75% of N through LMBR from *Chlorella* (T4) resulted in the maximum DMA in stem and roots which were significantly higher over RRF (*P* < 0.001). The leaf dry biomass in this treatment was nevertheless at par with RRF. However, this treatment recorded the significantly highest (*P* < 0.001) total dry matter in the vegetative parts than that in any of the other treatments. There was a marked decline in the DMA in stem and root in all the treatments receiving *Lyngbya* LMBR, although interestingly, none of them except T10 showed decline in leaf dry weight compared to RRF. This, however, resulted in overall significant decline in the total vegetative DMA, compared to RRF. Further, all the *Lyngbya* substitution treatments were found at par with respect to total DMA.

**Table 4:** **Effect of different LMBR treatments on dry matter accumulation in above-ground vegetative parts and roots of maize plant**.

**Treatments**	**Dry matter accumulation (g)**			
	**Stem**	**Leaves**	**Root**	**Total**
T1	82.00±4.95^cd^	79.92±4.55^bcde^	76.64±8.78^bc^	238.56±9.70^cd^
T2	86.45±9.02^c^	82.27±3.02^abc^	85.84±7.81^b^	254.56±12.71^bc^
T3	87.14±6.73^bc^	84.18±11.32^ab^	76.92±5.14^bc^	248.24±18.10^bc^
T4	109.14±13.57^a^	75.57±3.10^cde^	114.74±13.12^a^	299.45±15.10^a^
T5	97.61±11.34^b^	88.71±6.16^a^	72.16±6.00^c^	258.48±18.44^b^
T6	77.49±5.83^cd^	74.33±5.31^de^	75.78±11.15^bc^	227.59±17.78^d^
T7	81.08±6.76^cd^	81.81±7.83^abcd^	44.003.66^d^	206.89±8.16^e^
T8	73.13±7.16^d^	75.25±4.87^cde^	52.88±7.63^d^	201.27±14.52^e^
T9	73.73±10.90^d^	75.83±3.60^cde^	53.24±4.20^d^	202.80±12.88^e^
T10	79.25±6.40^cd^	73.28±5.29^e^	47.62±8.78^d^	200.15±17.25^e^

Values followed by different alphabets in columns are significantly different at p < 0.05 by LSD.

### Stress enzymes

**Figure 1:**
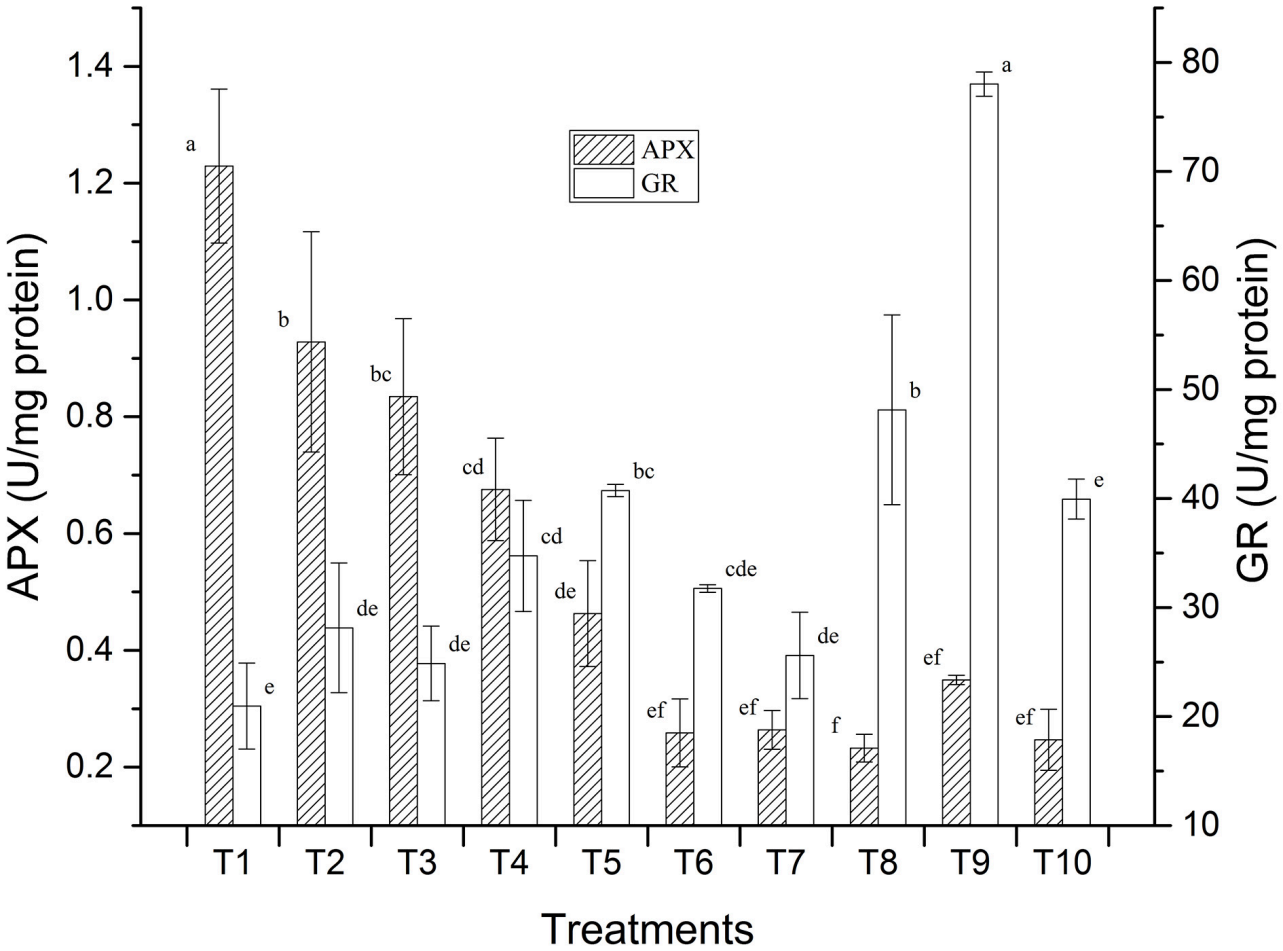
**Effect of LMBRs on activities of ascorbate peroxidase and glutathione reductase enzymes of maize at 60 DAS**.

Preliminary studies on LMBR treated plants also revealed positive results with respect to the stress enzymes studied which was carried out with a view to assess whether the application of the LMBRs on maize plants elicit any stress response. Two key enzymes, namely, ascorbate peroxidase (APX) and glutathione reductase (GR) activities were assayed (Figure 1). It was found that the treatment receiving no external nutrition recorded the highest APX activity, which was significantly higher than that in RRF (*P* = 0.011). Higher APX is suggestive of greater oxidative stress. All the other LMBR treatments had significantly lower activity of this enzyme (*P* ≤ 0.027) than RRF except in T3, which received N nutrition solely through LMBR of *Chlorella*. Interestingly, the APX activity decreased with decreasing dose of *Chlorella* LMBR. However, all the treatments employing LMBR of *Lyngbya* recorded significantly lower activity (*P* < 0.001) over RRF, which were at par with each other and thus was suggestive of decreased oxidative stress. GR activity was lowest in T1, which was, however, at par to RRF. Among the *Chlorella* treatments, only T5 recorded significantly higher values of GR activity over RRF (*P* = 0.015), while the rest were at par. All the *Lyngbya* treatments except that utilizing LMBR solely as N source recorded significantly higher GR activity over RRF, the highest being in T9, which was superior to all the other nine treatments (*P* < 0.001).

### Effect on yield attributes and grain yield

The length, width, fresh weight as well as the dry weight of the maize cobs with or without the outer husk remained statistically similar in all the LMBR treatments comparable to that in the RRF. Similarly, the same trend was found in number of kernel rows per cob and number of kernels (grains) per row, which determine the total number of grains formed (Table 5). Notably, T8 treatment that substituted 75% of chemical N by *Lyngbya* LMBR had significantly heavier grains compared to RRF (*P* = 0.008), which however was at par with all other LMBR treatments except in T7 where 100% of N was supplied by LMBR from *Lyngbya*. The highest grain weight (65.16 g plant^−1^) was measured in T10 where 25% of the total N was supplied by *Lyngbya* derived LMBR and rest by urea, which was closely followed by that (63.48 g plant^−1^) measured in T5 treatment where N was supplied to the plants in equal proportion through *Chlorella* LMBR and urea. However, both these treatments were found at par with other levels of substitutions with LMBRs from either of the algae and were also equivalent to RRF. The yield and its attributes were found the lowest in the absolute control treatment where no external nutrition was supplied.

**Table 5:** **Effect of different LMBR treatments on yield attributes and grain yield of maize**.

**Treatments**	**Grains dry weight per plant (g)**	**100 grains dry weight (g)**	**Length of kernel set on cob (cm)**	**No of kernels line on cob**	**No of kernels per line**
T1	40.99±6.11^b^	8.06±1.81^c^	13.73±2.90^b^	14.40±2.61^b^	30.47±0.90^b^
T2	61.21±10.84^a^	8.60±0.86^bc^	18.96±2.33^a^	16.00±0.00^ab^	32.13±14.10^ab^
T3	60.36±5.26^a^	9.63±0.87^ab^	17.82±1.69^ab^	16.80±1.10^a^	40.40±4.25^ab^
T4	59.20±3.58^a^	10.05±1.68^ab^	17.56±2.94^ab^	14.80±2.28^ab^	38.80±8.13^ab^
T5	63.48±4.45^a^	10.02±0.74^ab^	18.25±1.49^a^	16.00±1.41^ab^	39.87±3.20^ab^
T6	55.70±11.33^a^	9.94±1.38^ab^	15.67±3.46^ab^	15.60±0.89^ab^	34.40±7.95^ab^
T7	56.29±13.34^a^	9.11±0.86^bc^	16.61±2.85^ab^	16.40±1.67^ab^	36.20±6.30^ab^
T8	64.93±3.68^a^	10.68±0.72^a^	18.25±7.38^a^	15.60±0.89^ab^	32.87±13.01^ab^
T9	60.46±6.92^a^	10.05±1.32^ab^	17.62±1.81^ab^	15.60±1.67^ab^	39.53±5.74^ab^
T10	65.16±9.45^a^	9.67±0.91^ab^	18.91±1.96^a^	15.60±1.67^ab^	41.00±3.54^a^

Values followed by different alphabets in columns are significantly different at p < 0.05 by LSD.

### Effect on nutritional quality of maize

The carbohydrate, crude protein, and crude fat in maize grains were determined and are presented in Table 6. The carbohydrate content was the maximum where 75% of N was supplied through LMBR of *Chlorella*. This treatment was at par to all the other combinations of *Chlorella* LMBR, but was significantly superior to RRF (*P* = 0.018) as well as all other *Lyngbya* treatments (*P* ≤ 0.021). However, all the *Lyngbya* treated plants were at par with RRF treated ones. There was no change in crude protein content in grains due to either of the two LMBR treatments as compared to that in RRF. All the algal biomass treated plants were equivalent with respect to crude fat content, except T3 where 100% N was supplied through *Chlorella* derived LMBR. With respect to nutritional quality, it was found that there was no change in nitrogen content of the grains due to any of the LMBR treatments when compared to RRF. Compared to RRF, T3, and T10 had the significantly (*P* < 0.05) highest phosphorus content in the *Chlorella* and *Lyngbya* derived LMBR treatments, respectively. However, all the LMBR treatments were at par with each other with respect to P content in grains. Compared to RRF, there was significantly (*P* < 0.001) higher potassium uptake in grains in all the LMBR treatments except where N was entirely supplied through *Chlorella* derived LMBR. There was a marked increase in sodium as well as potassium content of grains in T10 treatment when compared to that in RRF.

**Table 6:** **Effect of different LMBR treatments on nutritional quality of maize**.

**Treatments**	**Carbohydrate (%)**	**Crude protein (%)**	**Crude fat (%)**	**Nitrogen (%)**	**Phosphorus (%)**	**Potassium (%)**	**Sodium (%)**
T1	57.19±2.06^cd^	8.73±1.46^b^	10.57±0.82^c^	1.40±0.23^b^	0.28±0.06^ab^	0.51±0.08^bc^	0.039±0.014^c^
T2	57.97±3.23^bcd^	9.02±0.91^ab^	10.63±1.30^bc^	1.44±0.15^ab^	0.18±0.05^b^	0.33±0.02^d^	0.043±0.010^c^
T3	60.68±3.67^abc^	11.27±3.76^a^	11.82±0.98^a^	1.80±0.60^a^	0.39±0.41^a^	0.27±0.10^d^	0.046±0.012^c^
T4	63.21±3.59^a^	9.85±0.87^ab^	11.26±0.78^abc^	1.58±0.14^ab^	0.28±0.03^ab^	0.44±0.09^c^	0.048±0.025^bc^
T5	61.49±4.78^ab^	9.68±1.20^ab^	11.26±0.78^abc^	1.55±0.19^ab^	0.28±0.05^ab^	0.56±0.04^ab^	0.044±0.004^c^
T6	60.47±1.54^abc^	9.62±1.02^ab^	11.30±0.97^abc^	1.54±0.16^ab^	0.35±0.12^ab^	0.58±0.06^ab^	0.047±0.004^bc^
T7	58.08±3.45^bcd^	9.59±2.36^ab^	11.05±0.85^abc^	1.53±0.38^ab^	0.36±0.14^ab^	0.58±0.11^ab^	0.044±0.005^c^
T8	57.02±4.06^cd^	9.31±0.75^ab^	11.75±0.59^ab^	1.49±0.12^ab^	0.30±0.06^ab^	0.52±0.02^abc^	0.042±0.006^c^
T9	57.24±1.84^bcd^	9.71±1.82^ab^	11.53±0.90^abc^	1.55±0.29^ab^	0.28±0.05^ab^	0.52±0.02^abc^	0.078±0.013^a^
T10	53.92±3.90^d^	10.28±2.47^ab^	11.07±0.75^abc^	1.64±0.40^ab^	0.41±0.22^a^	0.62±0.16^a^	0.062±0.004^b^

Values followed by different alphabets in columns are significantly different at p < 0.05 by LSD.

### Effects on physico-chemical properties of soil at harvest

The changes in physico-chemical properties brought out due to the various treatments are presented in Table 7. It was found that there was no significant difference in pH, EC, organic carbon, and available N content in the LMBR treated soil when compared to RRF treated ones. Other than the treatments supplying 100% (T3) and 75% (T4) N through *Chlorella* derived LMBR, all other treatments were at par to RRF with respect to available P content. The two aforesaid treatments were significantly (*P* ≤ 0. 006) better than all other treatments, but at par with each other. T4 and T5 in case of *Chlorella* based LMBR and T8 and T10 in case of *Lyngbya* based LMBR treatments recorded significantly lower available K in soil at harvest, while all other treatments were at par with RRF.

**Table 7:** **Effect of different LMBR treatments on physico-chemical properties of soil at harvest**.

**Treatments**	**Moisture (%)**	**pH**	**EC (dS/m)**	**Available N (g/kg)**	**Available P (g/kg)**	**Available K (g/kg)**	**Organic carbon (%)**
T1	15.89±1.65^a^	7.69±0.24^a^	1.14±0.12^b^	0.13±0.03^b^	0.03±0.01^bc^	0.39±0.06^a^	0.20±0.03^a^
T2	13.24±2.30^b^	7.47±0.17^a^	1.29±0.24^ab^	0.12±0.02^b^	0.05±0.02^b^	0.40±0.06^a^	0.15±0.04^b^
T3	13.68±1.82^ab^	7.58±0.15^a^	1.56±0.27^a^	0.13±0.05^b^	0.08±0.03^a^	0.39±0.11^a^	0.20±0.03^ab^
T4	13.78±0.95^ab^	7.59±0.22^a^	1.32±0.24^ab^	0.13±0.04^b^	0.08±0.02^a^	0.29±0.01^b^	0.18±0.03^ab^
T5	15.00±2.43^ab^	7.66±0.42^a^	1.19±0.20^b^	0.15±0.04^b^	0.03±0.02^bc^	0.29±0.05^b^	0.19±0.03^ab^
T6	13.83±3.75^ab^	7.63±0.21^a^	1.25±0.10^b^	0.15±0.03^b^	0.03±0.01^bc^	0.34±0.08^ab^	0.19±0.02^ab^
T7	13.64±1.43^ab^	7.63±0.26^a^	1.24±0.21^b^	0.16±0.10^b^	0.02±0.01^b^	0.31±0.09^ab^	0.16±0.06^ab^
T8	15.37±0.81^ab^	7.79±0.21^a^	1.32±0.38^ab^	0.41±0.24^a^	0.03±0.01^b^	0.36±0.08^b^	0.18±0.02^ab^
T9	13.44±1.39^ab^	7.76±0.23^a^	1.29±0.22^ab^	0.14±0.05^b^	0.02±0.00^b^	0.34±0.08^ab^	0.17±0.05^ab^
T10	14.50±1.22^ab^	7.76±0.39^a^	1.30±0.27^ab^	0.26±0.18^b^	0.04±0.02^bc^	0.29±0.04^b^	0.19±0.03^ab^

Values followed by different alphabets in columns are significantly different at p < 0.05 by LSD.

## Discussion

Among the LMBR from the two algal species, *Chlorella* derived one apparently was found to be more responsive in improving the plant height as no response was found through LMBR from *Lyngbya* when compared to RRF (Table 2). Nitrogen nutrition derived equally from both organic and chemical sources seemed to be the best balanced dose that sustained higher plant height relative to RRF starting from as early as 20 DAS till 80 DAS. It was observed that substitution of 100–50% of total N (T3–T5) through *Chlorella* LMBR treatments maintained the plant height at par with RRF or was even higher until 60 DAS. However, lowest rate of substitution in T6 (25%) brought about a diminution suggesting the role of the algal biomass toward sustained supply of nutrients or other active factors to the plants.

With respect to total DMA in the above ground vegetative parts and roots, contrasting results were found between LMBR from *Chlorella* and *Lyngbya* (Table 4). Whereas, 100 to 50% N substitutions through *Chlorella* biomass resulted in higher or equivalent DMA, similar levels of substitution by *Lyngbya* resulted in significant decrease when compared to that in RRF, suggesting the presence of one or more factor in the latter that are responsible for diminution of growth. Substitution of 25% N through either of the LMBR invariably brought a significant decrease in the aforesaid total DMA, connoting the requirement of *Chlorella* in higher amounts and confirming the retardant activity of LMBR from *Lyngbya* even at lower doses of substitution. The differential response between the two algal LMBRs might be on the account of their composition, which thus needs to be fully unraveled. This may indicate the presence of some toxic ingredients in *Lyngbya* species that negatively affect the plant growth. There are reports of toxicity of the microalgal extracts affecting the growth of plants (Romanowska-Duda and Tarczyñska, 2002) thus validating the argument (Grzesik and Romanowska-Duda, 2015) that untested algal residues should not be employed for treatment on plants.

Interestingly, there was no decrease in DMA in leaf due to the *Lyngbya* treatments except that in T10 where least level of substitution (25%) was done, which was also equivalent to all the other *Lyngbya* treatments. T4 fared the best among all the treatments for total DMA which could be primarily attributed to increase in stem and root biomass, while T5 formed the highest leaf biomass, which was although similar to that in RRF. The highest plant height and leaf biomass found in the LMBR treatment using biomass of *Chlorella* in equal proportion as chemical N (T5) at 60 DAS can be well-corroborated by the leaf chlorophyll index and net photosynthetic rate recorded during this time (Table 3), which were found to be the highest among all the *Chlorella* treatments and significantly superior to RRF. Notably, in spite of lower DMA in the vegetative biomass in all the *Lyngbya* treatments, compared to RRF, there was no significant decrease in net photosynthetic rate at the leaf level, as evident from its values recorded at 30 and 60 DAS (Table 3). Rather, it was significantly higher than all other treatments at 60 DAS. This treatment also showed the best chlorophyll index among all the *Lyngbya* LMBR treatments at all the times and was significantly higher than RRF before (60 DAS) as well as after tasseling stage (100 DAS) of the plant. Grzesik and Romanowska-Duda (2015) also similarly reported intensification of several metabolic processes such as net photosynthesis and other gas exchange parameters, stability of cytomembranes which eventually led to enhanced shoot and root dry weight of willow plants by application of sonicated biomass of two species of Cyanobacteria (*Microcystis* sp. and *Anabaena* sp.) and one green algae (*Chlorella* sp.).

Thus, the observations recorded on plant height, leaf chlorophyll index, net photosynthetic rate and above ground vegetative DMA in the treatments using LMBR from *Chlorella* suggest that any level of substitution resulted in either maintaining the growth parameters or improving it in some of the treatments. This possibly explained for the equivalent grain yield obtained from all the *Chlorella* derived LMBR treatments, compared to RRF. Besides, microalgae, which itself is photosynthetic, have all the plant essential elements in its biomass (Table 1) which are left in the residue after oil extraction and upon decomposition are available to the plants fertilized with it. Even though there was a detrimental effect on the total DMA by using LMBR from *Lyngbya* (Table 4), the maintenance of similar or higher photosynthetic rate leading to attainment of similar leaf biomass might have resulted in better partitioning of photosynthates to the grains, thus explaining for the similar grain yields despite the mentioned limitations. Evidently, even though the grain yield of maize was found similar to RRF in all the LMBR treatments, irrespective of the species of algae it was derived from, there were differences in the quality of grains due to the different treatments (Table 6). Whereas, there was no change in the protein content, T4 was nutritionally superior in terms of carbohydrate content, while T3 was superior with respect to fat and phosphorus content when compared over RRF. T10 was superior in terms of P, K as well as Na content when compared to RRF.

Strikingly, it was found that all the LMBR treatments except T3 had marked enhancement in the potassium content. Potassium has a prominent role in imparting stress tolerance in plants (Zörb et al., 2014). Another notable observation was that all the LMBR treatments exhibited a significantly longer tassel compared to that in RRF except that in T6 and T7, which were also numerically superior (Table 1). A longer tassel has been reported to be desirable under stress environment to ensure sufficient and extended pollen availability (Sofi, 2007). Even though the plants in the present experiment were not subjected to any specific stress, nevertheless, plants do experience biotic and abiotic stress, even at different times of the day during its growth and reproductive phase when grown under uncontrolled conditions (Layek et al., 2015). Such stress may be due to temperature extremities during day or night, incipient wilting during mid-day, variation in soil moisture, pest attack, etc., all of which could induce instantaneous production of reactive oxygen species (ROS). The results obtained from our studies on ROS scavengers like APX suggests that LMBRs may also be important for imparting tolerance under environmental stress and may be explored in the future on this aspect. The presence of bioelicitors in the LMBRs may also be explored which can perhaps trigger plant defense pathways in response to biotic and abiotic stresses. Such elicitors have been reported in other green algae like *Ulva lactuca* (Grzesik and Romanowska-Duda, 2015).

Evidently, no detrimental effect was found in the pH and EC of the soil due to the various LMBR treatments (Table 7). Although, there was no change in organic carbon content of soil, continuous application of the carbon rich LMBR may have a significant beneficial effect in the long run. The treatments employing 100% (T3) and 75% (T4) N through *Chlorella*-derived LMBR had a striking effect in improving available phosphorus in soil which may be ascribed to the improved microbial and associated biochemical activity of such carbonaceous materials (Anand et al., 2015). Interestingly, T3 also had the highest P content in the grains, all of which connote toward greater mineralization of soil phosphorus and also the higher mobilization of phosphorus to the grains of soil, which needs to be investigated in future. Grzesik and Romanowska-Duda (2014) recently reported that application of microalgal residues to corn plants significantly enhanced the activity of phosphatase enzyme, which is responsible for distribution of P in plants. The use of LMBR may also confer another advantage by preventing the loss of soil nutrients by way of leaching or volatilization, thus being a sustainable release nutrient source. Mulbry et al. (2005) reported that only 3% of algal N was present as plant available N in soil at the time of application of dried algal biomass (day 0). Similar to our studies, they also found that dried algal biomass was equivalent to fertilizer in supplying N and P to cucumber and corn. It was further conjectured that NH_3_ volatilization would not occur by use of dried algal biomass as manure in contrast to the use of chemical fertilizers.

Chemical solvent extraction is the most common method to effectively extract lipids from algae cells, but use of solvents like n-hexane and electric power, makes the cost of oil production high. In our case, LMBR was produced on a smaller scale for the purpose of conducting pot experiments. Other methods like wet extraction (Reddy et al., 2014), supercritical fluid technologies using CO_2_ (Halim et al., 2011), milking extraction methods (Kleinegris et al., 2010) are emerging as newer technologies for lipid extraction that would considerably economize on the cost and energy requirement. Even though efforts to extract the microalgal lipids and recover the solvent used has been recently demonstrated by using solar driven solvent extractor (Ghosh et al., 2013), nevertheless, it is extremely important that the algal residue obtained after extraction of the main product (oil) should be used in a biorefinery approach, which was the primary objective of the study. We found that the LMBRs deserved proper exploitation as fertilizer substitute as they are rich in plant nutrients. Considering the state of the art of algal production and post-harvest technology, the current production of biofuel from microalgae is technically feasible, but its economic feasibility is still a major challenge (Chiaramonti et al., 2015). The results of the present experiment applying the biorefinery concept to valorize the LMBR as a plant nutrient source is one of the ways to achieve economic sustainability of algal biofuel.

In view of promising results and due to the limited information available in the literature on this aspect of worldwide interest, further studies are required to evaluate the effect of the use of LMBRs on crop plants over the long term and understand the mechanism of action as well. The effects of LMBRs on soil microbial and biochemical properties, which have bearing on the nutrient cycle, also need to be studied in the future. Other active ingredients or anti-nutrients present in LMBRs also need to be characterized.

## Conclusion

In the present study, nitrogen-rich LMBRs of two microalgal species, namely, *Chlorella* and *Lyngbya* were utilized to substitute for the chemical nitrogen fertilizer requirement of maize plants in different proportions viz., 100, 75, 50, and 25%. The grain yield obtained using either of the LMBRs were equivalent to that under treatment employing RRF solely through chemical fertilizers. Even though LMBR of *Lyngbya* resulted in diminution of the stem and root biomass, there was no reduction observed in the formation of leaf biomass and photosynthetic rate as a result of which the translocation of assimilates from the source to sink was maintained and similar grain yields to that in RRF were obtained using it. Substitution of 75% of the total chemical N through *Chlorella* LMBR (T4) resulted in the highest DMA in above-ground vegetative biomass and roots and this treatment also recorded the highest grain carbohydrate content, all of which were significantly superior to that in RRF. Among the *Lyngbya* treatments, substitution at 25% level (T10) was found to be the best in terms of grain yield production as well as phosphorus and potassium content in grains, while carbohydrate, protein as well as fat content were found at par with RRF. Decreased APX enzyme activity, indicative of lower plant stress was also apparent in most of the LMBR treatments. There was also no detrimental effect of the various LMBR treatments on soil properties. Based on the results, it is concluded that both of the LMBRs can substitute, wholly or partially, the chemical nitrogen fertilizer without affecting the yield and quality of the maize crop, thereby reducing the usage of chemical fertilizers in agriculture and simultaneously making gainful use of the LMBRs generated during biodiesel preparation. The results obtained from the present study thus helps to valorize the LMBR in the commodity markets, thereby advancing toward the economic sustainability of algae production for biofuels.

### Conflict of interest statement

The authors declare that the research was conducted in the absence of any commercial or financial relationships that could be construed as a potential conflict of interest.

## Abbreviations

LMBR: lipid extracted microalgal biomass residues
RRF: recommended rate of fertilizer
CRD: completely randomized design
DMA: dry matter accumulation
APX: ascorbate peroxidase
GR: glutathione reductase
SSP: single super phosphate
MOP: muriate of potash
DAS: days after sowing
TVS: total volatile solid
DAP: di-ammonium phosphate
ROS: reactive oxygen species.
